# A case study of blast eye injury at work place

**DOI:** 10.4103/2321-3868.123076

**Published:** 2013-12-18

**Authors:** Prabhakar Srinivasapuram Krishnacharya

**Affiliations:** 1 Department of Ophthalmology, Jagadguru Sri Shivarathreeshwara Medical College and Hospital, Jagadguru Sri Shivarathreeshwara University, Mysore, Karnataka India; 2 57, 8th Cross, 4th Main Vinayaka Nagara, Mysore, 570 012 Karnataka India

**Keywords:** Corneal laceration, intra ocular foreign body, penetrating keratoplasty, scleral fixated intraocular lens

## Abstract

This case report aims at investigating whether two consecutive surgical settings would be beneficial in achieving postoperative success for the patient with blast eye injury. A 45-year-old male patient admitted on 17^th^ October 2011 with history of blast eye injury. Right eye examination revealed central corneal laceration with incarceration of lens matter, multiple foreign bodies also seen embedded in the eyelid margins and in the left cornea. Computed ocular tomography showed a retained intraocular foreign body (IOFB) in the right eye. Simultaneous corneal laceration repair and extraction of the ruptured lens performed as primary procedure under general anesthesia. Intraoperative posterior capsule loss was noticed with vitreous presentation. Anterior vitrectomy with removal of the IOFB was done. Foreign bodies were also removed from the left cornea. Penetrating keratoplasty (PK) with scleral fixated intraocular lens implantation executed 4 months later as secondary procedure. Visual acuity maintained at 6/24 in 2 years follow-up. In conclusion, two consecutive surgical settings has the advantage of calculating the intra ocular lens power.

## Introduction

Explosive ocular injuries at work place are commonly reported worldwide with an adverse impact on the economic status of the family members. It is estimated from the National Eye Trauma System Registry that 2.4 million eye injuries occur annually in the United States, about one-quarter of serious injuries emerge at the workplace.[1] Recently, Indian eye trauma registry system was started during International Society of Ocular Trauma-conference held at Jaipur in the year 2012 with a motive to prevent and to improve the safety standards in the work place. Low socioeconomic status and lack of proper education seem to be associated with lifetime risk for men than women in the work place.[2] More serious open globe injuries were reported under the influence of alcohol, mostly during the night times and weekend days.[3]

**Table 1:** 

Access this article online	
**Quick Response Code:**	**Website:** www.burnstrauma.com
	**DOI:** 10.4103/2321-3868.123076

Since most of the explosive injuries cause multiple structural damage of the eye, there is no consensus regarding the timing and method of management. The surgical management probably depends on the surgeon to tackle the emergency situation and the facilities available for the primary repair of the wounds. The goal is towards the reconstruction of the anterior segment as a first aid and referring further to posterior segment experts for vitreoretinal problems. The present case report aims at investigating the visual outcome and any complications arising from managing the case by two separate surgical procedures.

## Case report

A 45-year-old male patient admitted on 17^th^ October 2011 with history of blast injury at work place. Chief complaints were severe photophobia, blepharospasm, ocular pain, and loss of vision in the right eye. Examination revealed multiple charred open wounds on the face and the eyelids. Particles of sand and mud were seen embedded in and around the eyelids. Both the eyes were congested with swollen eyelids. Slit lamp examination of the right eye showed a full thickness stellate-shaped corneal laceration with iris and probably lens matter incarceration measuring 4–6 mm in the central cornea. The lacerated corneal edges were edematous and fragile. The anterior chamber was shallow and flocculent lens substance observed in partially reformed anterior chamber. Pupillary examination was insufficient due to clouding of the anterior segment structures with ruptured lens. Left eye examination revealed diffuse multiple foreign bodies mostly sand particles embedded in the superficial layers of the cornea with few abrasions. Traumatic mydriasis noted with normal anterior chamber and lens. The presence of light perception was doubtful in both eyes. Intraocular pressure could not be measured due to severe photophobia and blepharospasm. Computed ocular tomography revealed a medium sized intraocular foreign body (IOFB) located probably in the vitreous cavity of the right eye. No intraocular foreign bodies were seen in the left eye.

**Figure 1:**
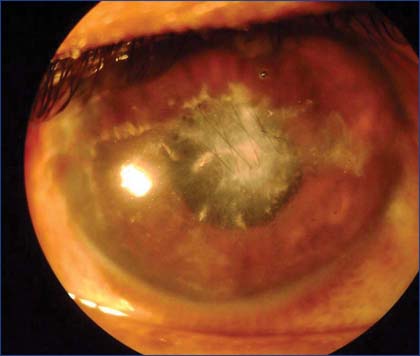
Right eye showing sutured healed corneal laceration 1 month post-operation.

**Figure 2:**
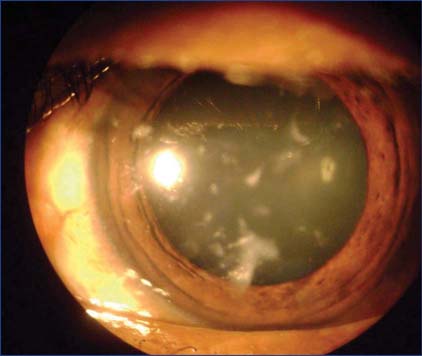
Multiple foreign bodies induced opacities and healed corneal abrasions in the left eye 1 month post-operation.

As a primary procedure corneal laceration was repaired and the ruptured traumatic cataract was extracted under general anesthesia. The eyelids and the conjunctival sac of the right eye irrigated with 5% povidone iodine as a prophylactic measure to prevent postoperative endophthalmitis. Multiple small to medium sized sand particles that embedded in the eyelid margins were removed. The surgical field was adequately exposed by eyelids skin stitches. Central corneal wound was repaired by continuous sutures using 10-0 monofilament Ethicon [Figure 1]. Through temporal scleral tunnel approach, hydrated lens matter was aspirated and giant posterior capsular rent was observed. Therefore, primary intraocular lens implantation was deferred to later dates. A fairly large sand particle was seen immediately floating in the anterior vitreous that was extracted by McPherson forceps. A mixture of air bubble and Ringer’s lactate was used to reform the anterior chamber and the scleral tunnel was closed with 10-0 Ethicon suture and a soft bandage contact lens was inserted. Multiple sand particles from the left cornea was simultaneously removed [Figure 2]. Postoperative B-scan ultrasound of the right eye revealed few hyper reflective echoes in the vitreous cavity. Intraocular pressure was 10 and 12 mmHg by rebound tonometer measured in the morning hours.

**Figure 3:**
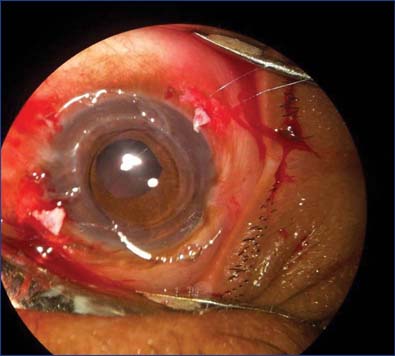
Intraoperative picture depicting trephined recipient’s cornea and scleral fixated intraocular lens.

**Figure 4:**
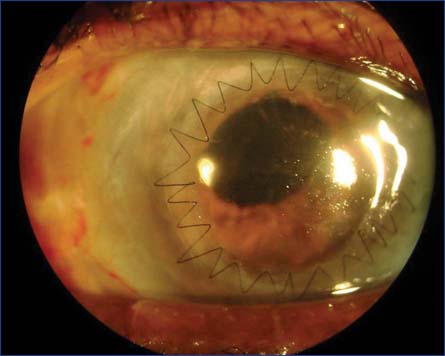
Image showing penetrating keratoplasty with scleral fixated intraocular lens implantation at first week post-operation.

Postoperative period was uneventful with an aphakic vision of hand movements in the right eye and 6/18 in the left eye. Prior to penetrating keratoplasty (PK) the right eye examination showed an irregular and dense central corneal opacification approximately measuring 4–5 mm obstructing the visual axis. Anterior chamber was optically empty with normally reacting pupil. Fundus examination was unremarkable. The best corrected visual acuity was 6/60 with normal intraocular pressure. Improved vision after the primary repair and decreased vision due to central leukomatous corneal opacity with normal posterior segment on ultrasound B-scan was the basis of the second surgery. In the month of February 2012, PK and scleral fixated intraocular lens implantation was performed in the right eye [Figures 3 and 4]. Postoperative vision improved to 6/60 in the right eye. During the 2 year follow-up no complications were noticed and the vision improved to 6/24 with surgically induced astigmatism of −4 diopters. The best corrected vision in the left eye was improved to 6/9.

## Discussion

In the present case report there was history of alcohol consumption during the working hours that might be correlated with severe degree of damage to the eye. Posture and proximity of the patient to the offending object could also explain the severity of the damage as our patient was very close to the explosion objects. Computed tomography (CT) of the right eye accurately localized the foreign body in the vitreous cavity that had travelled through the lens substance. CT scans demonstrate the presence of intraocular metallic and nonmetallic foreign bodies, and provide information regarding orbital fractures and lens injuries. Standard B-scan ultrasonography can also be used to detect metallic IOFBs, but the sensitivity is user-dependent. A small intraocular air pocket can be mistaken for an IOFB. Although contact ultrasonography is contraindicated in globes suspected of rupture, might be useful as an alternative imaging tool for the diagnosis of IOFB, if CT scan or magnetic resonance imaging (MRI) is not available on time as reported from the previous case study[4,5] MRI is contraindicated in the detection of suspected ferromagnetic retained intraocular foreign bodies, as it causes more intraocular tissue damage due to its migration.[6]

We decided first to repair the corneal laceration and extract the ruptured lens as primary procedure of choice. Primary intraocular lens implantation was not considered in this patient because of difficulty in calculating the axial length and corneal curvature due to collapsed anterior chamber posing problems for intraocular lens power calculation. Therefore, secondary intraocular implant was considered at a later stage until all postoperative inflammation subsided. Although reports from the previous study revealed no significant difference observed on final visual outcome between primary and secondary procedures of either cataract management with or without intraocular lens implantation.[7] Simultaneous traumatic cataract extraction with corneal laceration repair was significantly associated with faster visual rehabilitation with prevention of ruptured lens related complications as reported from the previous study[8]

Scleral fixated intraocular lens implantation was first described by Malbran *et al .*, in 1986.[9] Although the technique was associated with significant postoperative complications, it is safer procedure in eyes with complete loss of posterior capsule. Macular edema was frequently encountered problem that resolved in most of the patients which was not observed in the present case.[9] The author experienced good success rate with keratoplasty for managing injured blast victims that is coinciding with our patient.[10] Prognosis for the left eye seem to be good as there was no severe damage occurred except for the multiple foreign body induced superficial opacities and healed corneal abrasions that could cause visual disturbances such as glare and irregular astigmatism. Visual prognosis could be excellent if deep anterior lamellar keratoplasty be considered. The shortages of treatment of the present case report may by scleral fixated intraocular lens procedure involved use of 10-0 prolene, although advancements of suture less and glue less procedures are now being popular and advanced treatment modalities for secondary intraocular implantation. Surgical suturing technique followed was traditional type. Noncompliance of the patient was observed during the follow-ups due to lower economic conditions.

In conclusion, considering the management of the present case no complications were observed in the postoperative periods as the ruptured lens was completely extracted with corneal laceration repair. Computed ocular tomography provided accurate diagnosis for localization of IOFB. We found that secondary procedure has the advantage of finding out correct axial length and keratometric readings for intraocular lens power calculations which was difficult to achieve in the primary setting. Visual prognosis following the surgical treatments given to the right eye was better as the patient presented early. Occupational explosion eye injuries are avoidable through use of safety glasses and abstinence from alcohol at work place. There is a need to improve the safety standards at the working area and provide appropriate training to the employees by the concerned authorities.
